# Competence of teachers towards managing trauma among children with disabilities in Ghana

**DOI:** 10.4102/ajod.v13i0.1282

**Published:** 2024-02-23

**Authors:** Maxwell P. Opoku, Negmeldin Alsheikh, Daniel Miezah, Haseena Shah, Hala Elhoweris, Ashraf Moustafa

**Affiliations:** 1 Department of Special Education, College of Education, United Arab Emirates University, Al-Ain, United Arab Emirates; 2 Department of Education and Psychology, Faculty of Educational Foundations, University of Cape Coast, Cape-Coast, Ghana

**Keywords:** inclusive education, teachers, trauma management, students with disabilities, Ghana

## Abstract

**Background:**

Although trauma is one of the leading causes of behaviour problems among children with disabilities, there has been limited scholarly interest in trauma management within the discourse of implementation of inclusive education.

**Objectives:**

The Substance Abuse and Mental Health Services Administration (SAMHSA) trauma management model was used to study teachers’ awareness of trauma management among students with disabilities studying in regular classrooms.

**Method:**

A total of 271 teachers were recruited from two municipalities in the central region of Ghana to complete the Teacher Trauma Management Scale developed for this study. The data were analysed using confirmatory factor analysis, mean scores, multivariate analysis of variances, and linear regression.

**Results:**

The results showed teachers’ uncertainty towards trauma management, and a positive correlation was also found between the tenets of the study framework.

**Conclusion:**

The study concluded with a recommendation for contextual development of the curriculum to guide teacher training in trauma management.

**Contribution:**

Studies on trauma management within the discourse of implementation of inclusive education are scarce. This study extends the literature on inclusive education to teacher development to support trauma management among students with disabilities in regular schools.

## Introduction

Trauma refers to the overwhelming experience of a child that may adversely impact their decision-making and concentration on a given task (Phifer & Hull 2016; Substance Abuse and Mental Health Services Administration [SAMHSA] 2014). Trauma can result from various experiences, such as neglect, abuse, starvation, bullying, disasters, or witnessing deaths (Crosby 2015; Little & Maunder 2021; Maynard et al. 2019; Saleem, Howard& Langley 2022; SAMHSA 2014; Thomas, Crosby & Vanderhaar 2019). Discussions on appropriate ways to support individuals who have experienced trauma are essential because of the enormous consequences it can have (Berger & Marin 2021; Brunzell, Stokes & Waters 2016, 2019; SAMHSA 2014). For instance, trauma can have adverse effects on child development in the areas of academics, socialisation, and risk of mental health problems in the future (Berger & Martin 2021; Crosby 2015; Crosby, Howell & Thomas 2018; Little & Maunder 2021; Maynard et al. 2019; Saleem et al. 2022; SAMHSA 2014; Thomas et al. 2019). Children with disabilities who are unable to communicate their emotions and thoughts may be more affected by the consequences of trauma (Little & Maunder 2021; Schofield et al. 2021), making it difficult for them to seek assistance from their teachers. However, there is scarce literature on the ability of teachers towards managing trauma among children with disabilities. The purpose of this study was to assess the perceived competence of teachers towards trauma management among children with disabilities in inclusive schools in Ghana.

Scholarly interest in trauma-informed practices is increasing, particularly in Western countries such as Australia and the United States (e.g., Berger 2019; Berger & Martin 2021; Gubi et al. 2019; Harper & Neubauer 2021). Consequently, the SAMHSA model has been extensively utilised for researching school trauma management (Brunzell et al. 2016, 2019; Tweedie et al. 2017; Walker & Cox 2013). Two domains comprise this model: identifying and managing trauma, which inform support policies. The identification domain examines the impact of trauma on individuals, including events and experiences (SAMHSA 2014). Also, the trauma management domain covers the response of adults or caregivers to children experiencing trauma and their capacity to minimise the burden of trauma on individuals (SAMHSA 2014). The trauma management model is made up of six tenets: safety; trustworthiness and transparency; peer support; collaboration and mutuality; empowerment, voice and choice; and cultural, historical, and gender considerations.

The current study focusses on the trauma management domain, as research is urgently needed on actual teaching behaviour and perceived competence in addressing trauma in contexts such as Ghana. The six tenets of the trauma management domain were operationally defined. First of all, safety involves creating a safe and conducive learning environment (SAMHSA 2014). Trustworthiness and transparency entail teachers developing healthy relationships with their students and involving them in decisions pertaining to their well-being (SAMHSA 2014). Peer support can help teachers promote healthy relationships between students with disabilities and their typically developing peers. Collaboration and mutual support are critical when working with students with disabilities and their parents to enhance their emotional well-being (SAMHSA 2014). Empowerment entails providing a platform for students with disabilities to express themselves, encouraging them to embrace their situation, and working towards personal growth. Furthermore, it is essential to promote cultural, historical, and gender diversity while supporting policies that encourage the participation of all students (SAMHSA 2014).

Previous studies conducted using the SAMHSA model and other lenses, which were quasi-experimental, aimed at determining the effect of training on teachers’ skills in managing trauma for all children. Studies by Crosby (2015), Howard (2019), and Maynard et al. (2019) are some examples of such studies. Maynard et al. (2019) conducted an intervention study to demonstrate the impact of training teachers in school-wide behaviour support programmes. Based on the findings, the teachers demonstrated comprehension of trauma-informed practices and willingness to assist students susceptible to trauma. Additionally, Berger and Martin (2021) devised a structure to aid teachers in supporting students who are at risk of experiencing trauma. While the training programme has shown some success, it is recommended that teachers receive regular training and support services to assist their students better. Several studies have revealed that teachers often have limited knowledge of trauma management, and that schools lack trauma-related policies (Berger & Martin 2021; Phifer & Hull 2016). The findings of previous studies could be used to guide practices in western contexts where such studies were conducted. More so, the studies have focussed mainly on teachers’ knowledge about trauma management among typically developing children only. Indeed, in teaching children with disabilities, studies have reported that teachers in countries like Ghana face challenges in managing difficult classroom behaviour (e.g., Mensah et al. 2021). This highlights the need to extend the literature on trauma management to the African context in order to gather baseline information which could inform educational policies and practices.

In non-Western countries such as Ghana, implementing inclusive education is synonymous with creating learning opportunities for students with disabilities in regular classrooms (Sharma et al. 2017). Limited studies have been conducted globally on teachers’ knowledge of trauma management practices for children with disabilities (e.g., Schofield et al. 2021). To address this gap, the current study investigated the perceived competence of teachers in Ghana towards managing trauma among children with disabilities.

## Research context

Ghana is located in West Africa, with an approximate population of 30 million (Ghana Statistical Service 2021). Disability is defined as an impairment that limits individuals’ ability to see, walk, or perform daily tasks (Ghana Statistical Service 2021). It is estimated that 8% of the Ghanaian population lives with a disability (Ghana Statistical Service 2021). Despite the significant number of people with disabilities, they still face rejection, discrimination, and exclusion from important activities such as education and employment (Kassah, Kassah & Agbota 2014; Opoku et al. 2017). The attitude towards individuals with disability is a consequence of the intersection between disability and culture (Baffoe 2013; Mprah et al. 2016; Opoku et al. 2017, 2020). The onset of disability is linked to superstitions which contribute to their rejection by others (Baffoe 2013). The cultural stereotype towards individuals with disabilities is increasingly being challenged and there is ongoing advocacy towards creating awareness about aetiology of disability as well as promoting their acceptance in societies (World Health Organization 2011).

Ghana has taken positive steps towards inclusion by participating in the Salamanca conference and has started to educate children with disabilities in inclusive schools during the 2003–2004 academic year. This was further enhanced by promulgating the *Disability Act 715 in 2006* (Republic of Ghana 2006) and the ratification of the United Nations Convention on the Rights of Persons with Disabilities, which provided the impetus for educating children with disabilities in regular schools. Although progress has been made towards implementation of inclusive education, teachers continue to struggle to enact inclusive education for students with disabilities (Opoku et al. 2017, 2020; Sharma et al. 2013, 2017; Singal et al. 2015). However, limited attention has been given to teachers’ capacity to identify and manage trauma among children with disabilities who are at high risk of experiencing trauma because of cultural stereotypes and facing systemic barriers. The following research questions guided this study:

- What is the perceived competence of teachers towards trauma management among children with disabilities in Ghana?
- Which demographic variables can provide additional insights into trauma management among children with disabilities in Ghana?
- Which demographic variables can predict the perceived teacher competence towards trauma management among children with disabilities in Ghana?

## Research methods and design

### Study participants

In Ghana, education is structured as follows: early childhood (ages 4–6 years), primary (grades 1–6 [ages 6–12]), junior secondary (grades 7–9 [ages 12–15]), senior secondary (grades 10–12 [ages 15–18]), and tertiary education (Ministry of Education 2017). Education is compulsory through senior secondary. Inclusive education has been implemented at the national level, suggesting that all schools across the country are expected to teach every student in one classroom (Ministry of Education 2017).

**FIGURE 1:**
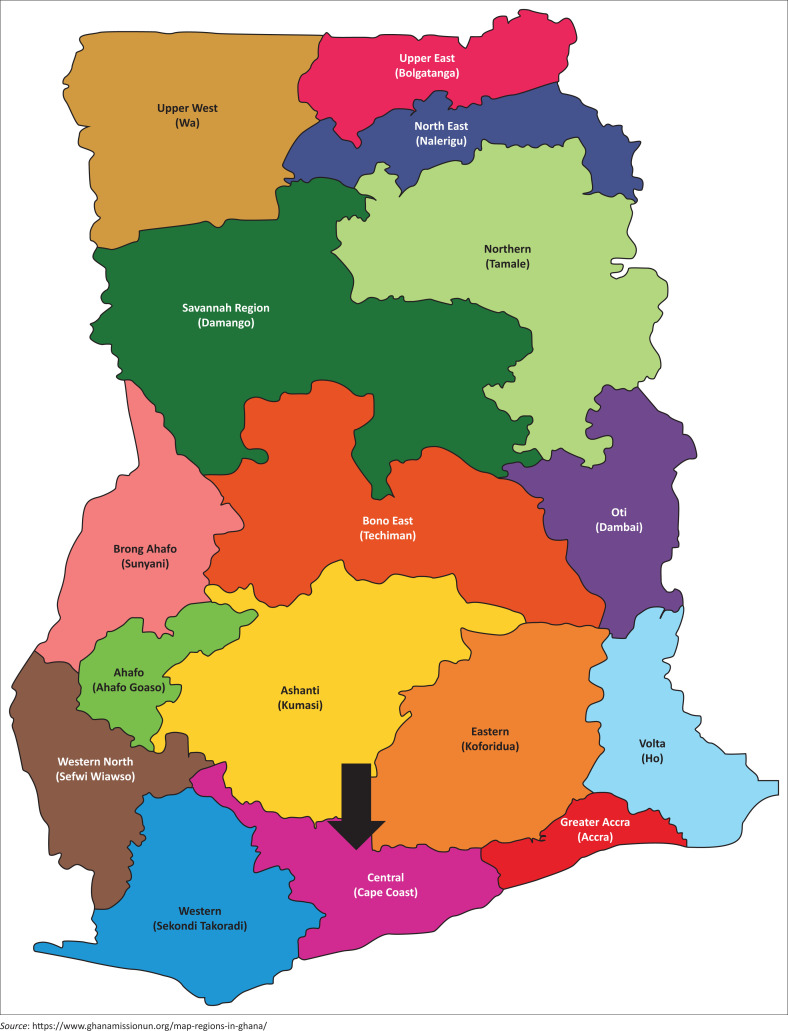
Map of Ghana showing the central region.

The target population for this study was teachers working in schools (early childhood to senior secondary) in Ghana. One of Ghana’s 21 administrative regions and terrestrial plains was conveniently selected for data collection, namely, Central region (see Figure 1). Within Central region, the Cape Coast Metropolitan district was further targeted for data collection. Out of an estimated population of 31 million in Ghana, 9.3% live in Central region, including 189 000 people living in Cape Coast Metropolitan district (Ghana Statistical Service 2021). Although information about the number of schools and enrolment in the region is scarce, it is where formal education began in Ghana. The public and private schools in the region are managed by the Ministry of Education Service, Ghana Education Service.

The teacher inclusion criteria were as follows: (1) teaching in a public or private school; (2) teaching at the early childhood, primary, intermediary, or secondary level; (3) teaching in an inclusive school; (4) familiarity with the concept of trauma; and (5) agreeing to participate in the study. As further detailed in the procedures section, 270 teachers from 18 schools met these criteria (Table 1).

**TABLE 1:** Demographic characteristics of study participants.

Category	Frequency (*N* = 270)	%
**Gender**		
Male	159	59
Female	111	41
**Age (years)**		
20–30	115	43
31–40	114	42
41 and above	41	15
**Marital status**		
Single	117	43
Married	153	57
**Number of children**		
None	99	37
1–3 Children	142	53
4 or more children	29	10
**Qualification**		
Bachelor’s	166	61
Master’s	104	39
**Level of teaching**		
Early childhood	19	7
Primary school	45	17
Junior Secondary	20	7
High school	186	69
**School type**		
Public school	220	81
Private school	50	29
**Training in trauma management**		
Yes	36	13
No	234	87
**Awareness of trauma policy**		
Familiar	90	33
Unfamiliar	180	67

### Instrument

The initial part focussed on background variables of participants, including gender, age, qualification, school type, level of teaching, marital status, number of children, awareness of trauma policy, and training in trauma management. The selection of demographic variables was based on a review of relevant literature (e.g., De Boer, Pijl & Minnaert 2011) and feedback from experts who evaluated the instrument prior to its implementation.

The second part was the Teacher Trauma Management Scale (TTMS), which comprises 53 items and six sub-scales (see Appendix 1). The instrument was anchored on a five-point Likert scale: (1) strongly disagree – (5) strongly agree. The development of this instrument followed a six-step approach: creation of the scale, defining constructs, clarifying purpose, item writing principles, validating the scale, and reporting on psychometric properties (Tay & Jebb 2017). Firstly, to create the framework, we followed a deductive approach by studying the existing literature on the relevant components. Secondly, we refined and clarified the components of the SAMHSA model which had already been defined for this study. Additionally, we reviewed previous studies that used this model to ensure its proper implementation in our study (Harper & Neubauer 2021; Saleem, Howard & Langley 2022; Schofield et al. 2021).

Thirdly, the scale’s purpose is to understand teachers’ perceived competence in managing trauma among their students with disabilities. Hence, since trauma is rarely discussed in the study context, the authors decided to simplify the components for completion by all teachers as step three. Fourthly, the item-writing principle encompasses the instrument’s appropriateness for implementation in different contexts. To achieve this, the first draft, which comprised 82 items, was given to five inclusive education and psychology experts to review and recommend its appropriateness for data collection. After considering the recommendations, a reduced draft, comprising 53 items, was deemed final for data collection: safety (items 1–12; *n* = 12), trustworthiness and transparency (items 13–21; *n* = 9), peer support (items 22–30; *n* = 9), collaboration (items 31–39; *n* = 9), empowerment (items 40–46; *n* = 7), and culture (items 47–53; *n* = 7).

Fifthly, for scale validation, content validation was performed by experts and statistical analyses. Additionally, the instrument was piloted on 20 teachers and yielded a reliability score of 0.78. The expert reviewers and some of the teachers who participated in the piloting provided some comments that were incorporated into the final draft used for data collection. The sixth step involved determining the psychometric properties of the newly developed scale. The results of the confirmatory factor analysis (CFA) and the reliability computation using Cronbach’s alpha (≥0.60) (Pallant 2020) were used to determine the appropriateness of the newly developed instrument.

### Procedures

This study and its protocols were approved by the Social Science Ethics Review Committee at United Arab Emirates University (ERSC_2022_585). Permission was also sought from the Cape Coast Ministry of Education Municipal Office. Afterward, the authors randomly selected the schools invited to participate. Random sampling was deemed appropriate as the intention was to ensure that teachers from diverse backgrounds participated in this study (Ary et al. 2019). The school leaders were sent formal invitations, and those who consented were included. Out of the 30 schools invited to participate in this study, 18 schools, representing 60%, agreed to participate in our study. An information statement explaining the study was forwarded to the teachers, followed by an email with a Google Form link to the survey.

The participants completed the instrument in English, and the data were collected between April 2022 and November 2022. The data collection was prolonged as it took time for teachers to respond to the survey. The email inviting the teachers provided information about the study, its objectives, their rights, and the confidentiality of the information collected. Consent was implied if the participants read the information statement and proceeded to participate in the study. No reimbursement was offered to the participants. They were assured that their school name, location, or identifiable information would not be used to report the study.

### Data analysis

The online data were transferred from Google Forms to Microsoft Excel for cleaning before transferring them to Statistical Package for the Social Sciences (SPSS) (IBM, Chicago, Illinois, USA) version 28 for analysis. The initial computation of the Kolmogorov–Smirnov and Shapiro–Wilk tests were not significant, thus supporting the data normality assumption for parametric analysis of the TTMS total scale score and six sub-scale scores. Following this, CFA was conducted to understand the underlying structure of the TTMS. Next, the appropriateness of the scale was determined using the following fit indices and cut-offs indicating a good model fit: a chi-square with significance *p* > 0.05, a comparative fit index (CFI) and a Tucker–Lewis index (TLI) value of at least 0.90, a root square error of approximation (RMSEA) and a standard root mean square residual (SRMR) value <0.08, and a regression weight of at least 0.50 (Byrne 2016). Moreover, the relationships between the six subscales were noted and interpreted as follows: small (0.10–0.30), moderate (0.31–0.50), and large (at least 0.51) (Pallant 2020).

Following this, we proceeded to answer the research questions. For research question 1, the mean scores of the TTMS total scale score and six sub-scale scores were computed to explore teachers’ perceived competence in adopting trauma management. Since the instrument was anchored on a point scale, a composite mean of 4 (sum mean divided by the number of items) was interpreted as adequate perceived knowledge towards trauma management.

For research question 2, association between demographic variables and trauma management was calculated for total scale and at sub-scale level. In relation to the total scale, t-tests (demographic with two levels) and analysis of variances (demographic variables with at least three levels) were calculated to explore the relationship between demographic variables and total management scale. Homogeneity of variances was observed to ensure that they were not violated.

In relation to the sub-scales, multivariate analysis of variance (MANOVA) was used to explore the association of teacher demographic characteristics with the combined dependent variables (i.e., the six sub-scale scores). Given that the latent variables measured different aspects of the same construct, it was appropriate to employ MANOVA to observe the combined and individual differences by the background variables. To reduce the risk of Type 1 error (guiding against making wrong conclusions), Pallant (2020) recommended a more stringent alpha value. Therefore, a Bonferroni-adjusted alpha level of 0.01 (i.e., 0.05 divided by the number of comparisons being made) (Pallant 2020) was set as the baseline for determining whether there were differences among the participants.

For research question 3, linear regression was computed to understand the contribution of demographic variables to the variance in teachers’ perception of competence in trauma management. Preliminary assessments ensured that the following assumptions were not violated: normality, linearity, multicollinearity, and homoscedasticity (Pallant 2020).

### Ethical considerations

Ethical clearance to conduct this study was obtained from United Arab Emirates University the Social Science Ethics Review Committee (No. ERSC_2022_585).

## Results

### Confirming the underlying structure of teacher trauma management scale

The 53-item TTMS (see Appendix 1) was validated using CFA, and iterated deletion of erroneous covariances improved the fit indices. Eighteen items were supported in this study and yielded the following fit indices: chi-square = 2.19 (minimum discrepancy function (CMIN]) = 269.82, df = 120, *p* = 0.001), CFI = 0.91, TLI = 0.90, RMSEA = 0.07, and SRMR = 0.05. In addition, each of the items had a regression weight of at least 0.50.

Configural invariance was estimated to ascertain whether the factor structure was valid across different demographics, such as gender (chi-square = 1.90 [CMIN = 1139.68, df = 600, *p* = 0.001], CFI = 0.90, TLI = 0.87, RMSEA = 0.03, and SRMR = 0.05) and school type (public vs. private school), confirming the underlying factor structure of the TTMS.

**FIGURE 2:**
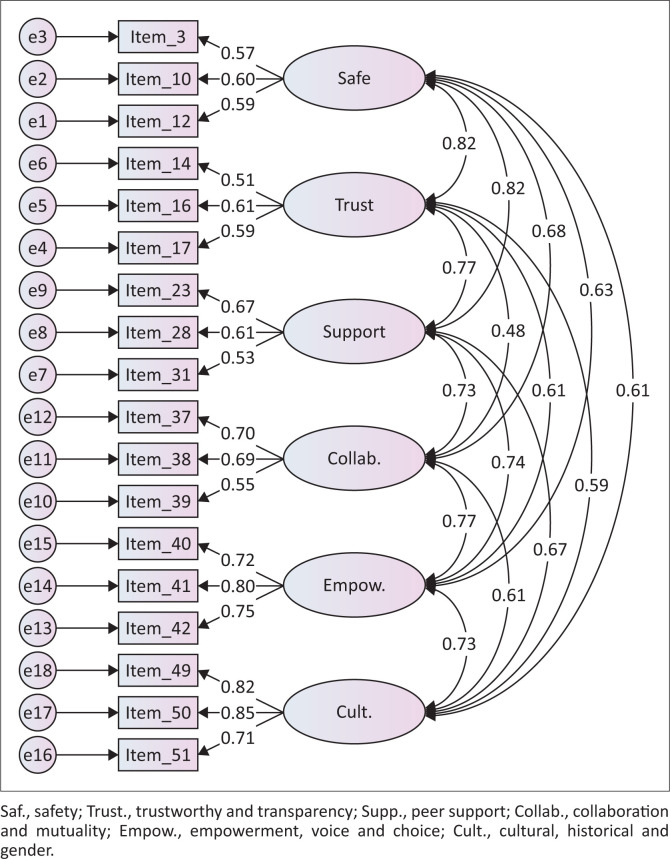
Summary of confirmatory factor analysis for teacher trauma management scale.

The reliability of TTMS using Cronbach’s alpha was 0.89, and the sub-scales yielded the following scores: safety = 0.61, trustworthiness and transparency = 0.60, peer support = 0.63, collaboration = 0.67, empowerment = 0.80, and culture = 0.83. As shown in Figure 2, correlation among the sub-scales was strong, ranging from 0.48 to 0.82.

### Perceived teacher competence

The overall mean score for teachers’ perceived competence to manage trauma among their students with disabilities was 3.25 (SD) and were as follows for the six subscales: safety (M = 3.12, SD = 0.89), trustworthiness and transparency (M = 3.17, SD = 0.77), peer support (M = 3.25, SD = 0.85), collaboration (M = 3.16, SD = 0.93), empowerment (M = 3.35, SD = 0.96), and culture (M = 3.43, SD = 0.98).

### Differences among teachers in trauma management

Association between demographic variables and trauma management was computed. The differences were calculated for total management (see Table 2) and at sub-scale levels (see Table 3). Multivariate analysis of variances were computed to measure differences between participants on trauma management (see Table 3). The results showed differences between participants on two demographic variables: marital status and training in trauma management. As for marital status, no significant difference was observed among the participants in the combined dependent variables, *F* (6, 263) = 1.52, *Wilks’ Lambda* = 0.97, *p* = 0.17, with a small effect size, *partial eta squared* = 0.03. Individually, there were differences between the participants in terms of safety, with married individuals rating themselves higher on safety compared to those who identified as single. This difference was statistically significant with a small effect size (F[1, 268] = 8.78, *p* = 0.003, *partial eta squared* = 0.03). The mean scores showed that married participants had an average safety rating of 3.26 (SD = 0.82). In contrast, single participants had an average safety rating of 2.94 (SD = 0.96).

**TABLE 2:** Association between demographic variables and trauma management.

Categories	Mean	SD
**Gender**		
Male	3.25	0.75
Female	3.25	0.59
*t*	−0.06	-
*Effect size*	0.007	-
**Age (years)**		
20–30	3.14	0.71
31–40	3.33	0.62
41 and above	3.33	4.71
*F*	2.36	-
*Effect size*	0.02	-
**Marital status**		
Single	3.13	0.72
Married	3.34	0.65
*t*	−2.56**	-
*Effect size*	0.30	-
**Number of children**		
None	3.26	0.67
1–3 Children	3.20	0.69
4 or more children	3.43	0.76
*F*	1.36	-
*Effect size*	0.01	-
**Qualification**		
Bachelor’s	3.28	0.67
Master’s	3.20	0.73
*t*	0.93	-
*Effect size*	0.11	-
**Level of teaching**		
Early childhood	3.46	0.82
Primary school	3.38	0.53
Junior Secondary	2.99	0.61
High school	3.22	0.71
*F*	2.26	-
*Effect size*	0.03	-
**School type**		
Public school	3.22	0.72
Private school	3.36	0.55
*t*	−1.24	-
*Effect size*	0.19	-
**Training in trauma management**		
Yes	3.47	0.68
No	3.21	0.69
*t*	2.11*	-
*Effect size*	0.38	-
**Awareness of trauma policy**		
Yes	3.24	0.67
No	3.22	0.70
*t*	0.78	-
*Effect size*	0.10	-

* , *p* ≤ 0.05;

** , *p* < 0.01.

**TABLE 3:** Multivariant analysis of variance showing the difference between participants.

Categories	Wilks’ Lambda	MAN. F	ANOVA F					
			Saf.	Trust.	Supp.	Collab.	Empow.	Cult.
**Gender**								
Male	-	-	3.12 (0.90)	3.09 (0.93)	3.27 (0.89)	3.16 (0.90)	3.36 (1.00)	3.47 (1.00)
Female	-	-	3.14 (0.89)	3.27 (0.75)	3.23 (0.80)	3.16 (0.98)	3.32 (0.92)	3.38 (0.96)
*F*	0.98	1.04	0.03	2.82	0.10	0.002	0.12	0.63
*Effect size*	-	0.02	0.01	0.01	0.001	0.001	0.001	0.002
**Age (years)**								
20–30	-	-	3.03 (0.92)	3.06 (0.92)	3.15 (0.92)	3.05 (0.93)	3.24 (0.93)	3.32 (1.01)
31–40	-	-	3.20 (0.82)	3.25 (0.76)	3.34 (0.76)	3.24 (0.93)	3.43 (0.98)	3.50 (0.93)
41 and above	-	-	3.20 (0.50)	3.24 (0.76)	3.31 (0.89)	3.24 (0.94)	3.42 (1.01)	3.55 (1.03)
*F*	0.98	0.43	1.22	1.63	1.42	1.33	1.18	1.31
*Effect size*	-	0.01	0.009	0.01	0.01	0.01	0.009	0.01
**Marital status**								
Single	-	-	2.94 (0.96)	3.07 (0.92)	3.15 (0.93)	3.03 (0.49)	3.25 (1.01)	3.33 (0.99)
Married	-	-	3.26 (0.82)	3.24 (0.81)	3.33 (0.78)	3.25 (0.89)	3.43 (0.93)	3.51 (0.49)
*F*	0.97	1.52	8.78*	2.59	2.89	3.76	2.37	1.98
*Effect size*	-	0.03	0.03	0.01	0.01	0.01	0.009	0.007
**Number of children**								
None	-	-	3.07 (0.91)	3.14 (0.85)	3.27 (0.85)	3.14 (0.97)	3.40 (0.98)	3.53 (0.89)
1–3 Children	-	-	3.14 (0.87)	3.15 (0.84)	3.17 (0.84)	3.14 (0.91)	3.28 (0.96)	3.34 (1.10)
4 or more children	-	-	3.25 (0.98)	3.36 (1.02)	3.60 (0.89)	3.31 (0.89)	3.51 (0.93)	3.57 (1.14)
*F*	0.96	0.94	0.49	0.77	3.09	0.43	0.90	1.41
*Effect size*	-	0.02	0.004	0.006	0.02	0.003	0.007	0.01
**Qualification**								
Bachelor’s	-	-	3.12 (0.87)	3.15 (0.86)	3.26 (0.84)	3.19 (0.88)	3.44 (0.92)	3.51 (0.93)
Master’s	-	-	3.13 (0.93)	3.19 (0.87)	3.25 (0.87)	3.12 (1.01)	3.19 (1.01)	3.30 (1.05)
*F*	0.97	1.55	0.02	0.13	0.004	0.38	4.66	2.74
*Effect size*	-	0.03	0.001	0.001	0.001	0.001	0.02	0.01
**Level of teaching**								
Early childhood	-	-	3.07 (0.87)	3.25 (0.92)	3.37 (1.05)	3.65 (0.93)	3.74 (0.91)	3.70 (1.07)
Primary school	-	-	3.36 (0.70)	3.19 (0.77)	3.36 (0.70)	3.30 (0.72)	3.56 (0.64)	3.52 (0.76)
Junior Secondary	-	-	2.92 (0.94)	2.82 (1.07)	3.03 (0.82)	2.90 (1.02)	2.93 (1.18)	3.32 (1.06)
High school	-	-	3.09 (0.93)	3.19 (0.86)	3.24 (0.87)	3.10 (0.95)	3.30 (0.99)	3.40 (1.01)
*F*	0.92	1.18	1.53	1.21	0.82	2.93	3.17	0.77
*Effect size*	-	0.03	0.02	0.01	0.009	0.03	0.03	0.009
**School type**								
Public school	-	-	3.11 (0.93)	3.17 (0.89)	3.23 (0.87)	3.13 (0.95)	3.31 (1.00)	3.39 (1.01)
Private school	-	-	3.21 (0.72)	3.15 (0.77)	3.37 (0.75)	3.27 (0.83)	3.51 (0.96)	3.63 (0.82)
*F*	0.99	0.65	0.52	0.02	1.07	0.92	1.81	2.42
*Effect size*	-	0.02	0.002	0.001	0.004	0.003	0.007	0.009
**Training in trauma management**								
Yes	-	-	3.49 (0.83)	3.48 (0.79)	3.52 (0.79)	3.31 (0.81)	3.51 (1.01)	3.52 (1.03)
No	-	-	3.07 (0.89)	3.12 (0.87)	3.21 (0.86)	3.14 (0.95)	3.14 (0.96)	3.42 (0.97)
*F*	0.96	1.68	7.13**	5.55***	4.03	1.16	1.16	0.32
*Effect size*	-	0.04	0.03	0.02	0.02	0.004	0.004	0.001
**Awareness of trauma policy**								
Yes	-	-	3.17 (0.92)	3.24 (0.80)	3.34 (0.83)	3.22 (0.88)	3.36 (0.92)	3.42 (0.98)
No	-	-	3.10 (0.88)	3.13 (0.89)	3.21 (0.86)	3.13 (0.95)	3.34 (0.99)	3.44 (0.98)
*F*	0.99	0.53	0.35	0.96	1.51	0.62	0.03	0.01
*Effect size*	-	0.01	0.001	0.004	0.006	0.002	0.001	0.001

Note: All data in brackets represent standard deviation.

Saf., safety; Trust., trustworthy and transparency; Supp., peer support; Collab., collaboration and mutuality; Empow., empowerment, voice and choice; Cult., cultural, historical and gender.

* , *p* = 0.003;

** , *p* = 0.008;

*** , *p* = 0.01,

In relation to training in trauma management, no difference was found between participants on the combined dependent variables, F (6, 263) = 1.68, Wilks’ Lambda = 0.96, with a small effect size, partial eta squared = 0.04. Individually, the difference was found between participants on safety (F [1, 268] = 7.13, *p* = 0.008, with a small effect size, *partial eta squared* = 0.03) and trustworthy (F [1, 268] = 5.55, *p* = 0.01, with a small effect size, *partial eta squared* = 0.02). With regards to safety, the mean scores showed that those who indicated they had received training (M = 3.49, SD = 0.83) in trauma management rated themselves higher compared to those who indicated otherwise (M = 3.07, SD = 0.89). Similarly, on trustworthy, participants who indicated they had received training (M = 3.48, SD = 0.79) in trauma management rated themselves higher than those who indicated they had not received any training (M = 3.17, SD = 0.86).

### Predictor of trauma management

The demographic variables were regressed on the total TTMS (see Table 4). The contribution of the demographic variables in the variance was 6%, *F* (9, 260) = 1.92, *p* = 0.05. However, only teachers’ marital status (b = 0.18, *p* = 0.01) significantly contributed to the variance in trauma management.

**TABLE 4:** Regression of demographic variables on trauma management.

Categories	Uns. Beta	SE	Stan. Beta	t	*p*
Gender	−0.34	1.58	−0.01	−0.22	0.83
Age	0.75	1.24	0.04	0.61	0.54
Marital status	4.44	1.78	0.18	2.49	0.01**
Number of children	−1.15	1.35	−0.06	−0.85	0.40
Qualification	−1.59	1.58	−0.06	−1.00	0.32
Level of teaching	−1.288	0.94	−0.104	−1.37	0.172
School type	1.26	2.06	0.04	0.61	0.54
Training in Trauma management	−5.33	2.25	−0.15	−2.37	0.07
Awareness of trauma policy	1.55	1.84	0.06	0.84	0.40

SE, standard error.

** , *p* < 0.01.

## Discussion

Trauma is considered an onset of challenging behaviours among children with disabilities (Hoch & Youssef 2020). This study was conducted against the backdrop of the scarcity of research on trauma management within the discourse of behaviour management. The study presents a valid instrument that could be used to measure the perceived competence of teachers towards managing trauma among students with disabilities. The instrument was subjected to rigorous validation and thus could be used by future researchers in African or similar non-western contexts. Trauma management among children with disabilities is unresearched in the African context. Notably, efforts towards implementing inclusive education have stalled (Singal et al. 2015), with teachers lacking the requisite teaching skills to support students with disabilities in classrooms (Mprah et al. 2016). Thus, there is an urgency to develop insight into skills required by teachers before they can respond appropriately to classroom trauma. The study has contributed to the literature by developing and validating an instrument that could be used to gather baseline data in other African contexts.

Trauma management is believed to be a complex phenomenon and, thus, requires a broad or supportive lens (Perry & Daniels 2016; Schofield et al. 2021). The study findings showed positive and significant correlations among all six tenets of trauma management (safety, trustworthiness and transparency, peer support, collaboration, empowerment, and culture). The relevance of the components of the SAMHSA model has been supported in previous studies on children with disabilities (Schofield et al. 2021) and general children (Perry & Daniels 2016). In inclusive classrooms, teachers consistently exclude children with disabilities, mainly because of their challenging behaviours (Sharma et al. 2013, 2017). As part of the effort towards promoting their education in classrooms, teacher training programmes could be developed based on the tenets of the SAMHSA. However, stakeholder engagements could be organised to discuss the components of the trauma training model before its implementation or reflected in teacher training curriculum.

The study findings provided information about teachers’ perceived competence towards trauma management in a non-western context. Specifically, the mean scores showed the participants’ neutrality toward trauma management. This suggests that participants who took part in this study might be unsure of the best ways to manage trauma among children with disabilities. This finding partly agrees with previous studies that reported teachers’ lack of knowledge regarding trauma management among general children in schools (Berger 2019; Berge & Martin 2021; Brunzell et al. 2019; Crosby 2015). The findings support Schofield et al.’s (2021) and Little and Maunder’s (2021) suggestions that studies on trauma management among children with disabilities in regular schools have not attracted scholarly interest. Countries such as Ghana are struggling to implement inclusive education (Ministry of Education 2017; Singal et al. 2015), and, as such, teachers must be equipped with the requisite knowledge of trauma management. In this way, they would be better positioned to support students with disabilities who are consistently at high risk of marginalisation or exclusion from school. Policymakers could consider engaging teacher educators on incorporation of trauma management in the teacher training curriculum.

One demographic variable that impacted trauma management was marital status. The computation of MANOVA and regression showed the importance of marital status in trauma management. It seemed married participants were more likely to create a safe learning environment for students with disabilities compared to single participants. This finding agrees with a previous study that reported the influence of marital status on inclusive teaching practices (Nwosu et al. 2020). In inclusive education research, it has been inferred that married teachers might be experienced in human relationships and raising children (Nwosu et al. 2020). This experience has been argued to be vital to enabling them to become better teachers of children with disabilities compared to those who are unmarried. Such inferences could hold for the participants of this study, who may have gained experience that enabled them to create an appropriate learning environment for children with disabilities. There is the possibility that married teachers might have children or have experiences living with their spouse. Consequently, they have developed useful skills to create a supportive learning environment and develop relationships with children with disabilities. In the literature, teachers struggle to create a safe learning environment as well as develop relationships with children with disabilities (Mensah et al. 2021; Sharma et al. 2013, 2017; Singal et al. 2015). This study seemed to show that married teachers could have skills that they have acquired through their familial experiences. However, the findings reported here are inconclusive, and future studies should explore how married teachers create supportive learning environments for such students.

Training in trauma management seems to impact teachers’ perceived competence toward implementing trauma-informed practices for children with disabilities. Specifically, the findings showed that those who indicated that they had received training in trauma management seemed to have more knowledge about providing a safe learning environment for children with disabilities than those who indicated otherwise. In inclusive education literature, a large body of literature has reported a synergy between training and knowledge acquisition to support classroom teaching practices (De Boer et al. 2011; Sharma et al. 2013, 2017). In the trauma literature, it has been reported that teacher training improves their trauma management skills (Berger & Martin 2021; Maynard et al. 2019). It is apparent that in the Ghanaian context, if teachers are trained in trauma management, they can create a safe learning environment for children with disabilities at risk of trauma. However, with participants generally ambivalent on each of the tenets, there is a need for more research to develop an in-depth understanding of the training needs of teachers.

Generalising the study findings is impossible because of numerous limitations. Firstly, the study focussed on teachers in both public and private schools in one of the 16 regions in Ghana. Hence, the interpretation of the findings was limited to the experiences of the teachers who participated in the study. However, Ghana is a multicultural society with teachers from diverse backgrounds teaching in public and private schools subjected to common regulatory standards, curriculum, and teacher recruitment. Thus, the teachers’ experiences in the included schools could mirror those outside the scope of this study. Secondly, the data were collected virtually, with teachers providing their responses digitally. Thus, the researchers were unable to ask follow-up questions or seek clarification. Future studies could also use qualitative methods to develop deep insights into the trauma management practices implemented by teachers in Ghana. Thirdly, the participants were recruited by the principals or the school administration. Thus, bias is possible, as the school administration could only share the link with teachers who might provide favourable responses. However, since teachers were provided with the information statement, the responses used in reporting this study could reflect the teachers’ perceived competence. Finally, this study focussed on trauma without looking at the capacity of teachers to identify and recognise traumatic events. Future studies could ascertain the relationship between trauma identity and management among teachers.

This study explored teachers’ perceived knowledge of trauma management among students with disabilities in regular classrooms in Ghana. This study was conducted because of the interlock between trauma and behavioural problems among children with disabilities (Little & Maunder 2021). The study findings may be considered in future educational reforms in Ghana. The transplantation of inclusive education policies, especially from Western contexts, has not helped non-Western countries strive to implement inclusive education (Kalyanpur 2014). This study showed that teachers were ambivalent on the tenets of the SAMHSA. There probably is the need for teacher training to improve their knowledge towards trauma management. However, local studies and engagement should drive the curriculum, which should be used for teacher training. For instance, policymakers’ leading efforts toward implementing inclusive education may organise regional meetings with stakeholders, such as parents, teachers, educators, and opinion leaders, to deliberate on the scope and best support strategies using the SAMHSA model as a conceptual model. These deliberations can help develop appropriate evidence to drive trauma policies and guidelines on management strategies that teachers can adopt. The deliberations could also shed light on formalising professional development in trauma management for in-service teachers. Students with disabilities are being denied accessible education, and steps to equip teachers to address behavioural problems could be essential in efforts to promote accessible education for all.

## Appendix 1

**Table:**

1	I am well trained to manage trauma among students with disabilities.
2	I understand trauma and its effects on children with disabilities.
3	I understand the emotional needs of children with disabilities who are traumatised.
4	I know the causes and triggers of trauma among children with disabilities.
5	I know when children with disabilities are traumatised.
6	I see myself as a good teacher for children with disabilities.
7	I can manage trauma among children with disabilities in classrooms.
8	I can help students with disabilities overcome trauma.
9	I know what to do to enable students with disabilities feel safe.
10	I can manage negative behaviours among children with disabilities associated with trauma.
11	I can create a safe and welcoming classroom for students with disabilities.
12	I have support plan for students with disabilities who are traumatised.
13	I am aware of policies and guidelines regarding dealing with students who have trauma.
14	I involve students with disabilities in decision-making on trauma management.
15	I see myself as a democratic teacher who listens to grievances of students with disabilities.
16	I know how to develop relationship with students with disabilities who are traumatised.
17	There is a trauma management policy that is known and accessible to all.
18	I contributed significantly in the development of the trauma policy and practice.
19	I can explain and make students with disabilities aware of trauma policy used in schools
20	I can make students with disabilities feel important and loved by all.
21	My students feel safe in the school classroom to share their problems related to trauma.
22	I can develop friendship among students with disabilities.
23	I can train students with disabilities to express and manage their feelings.
24	I can create an activity for all students with disabilities to share ideas and support each other.
25	I can train students with disabilities to cope with trauma.
26	There is a method for every student with disability to deal with stress.
27	I can train students with disabilities to deal with trauma for social acceptance.
28	I can invite parents to be involved in trauma management plans.
29	I know how to initiate conversations with parents on trauma management.
30	I know how to get parents to make input into trauma management.
31	The school environment is trustworthy to work with families and students on trauma related issues.
32	I can collaborate with students with disabilities to manage trauma.
33	I know how to empower students with disabilities to be in control and manage their behaviours.
34	I know how to interact with others to manage trauma among students with disabilities.
35	There is regular in-service training on trauma management for all teachers.
36	There is room for open discussion on trauma management among teachers.
37	I can befriend students with disabilities to manage trauma and difficulties.
38	I can encourage other peers to support trauma management for students with disabilities.
39	The school has a collaborative environment to support students and families on the issues of trauma.
40	I can educate students with disabilities to accept themselves.
41	I can empower students with disabilities to think positively.
42	I can give voice to students with disabilities who are affected by trauma.
43	I can ask students with disabilities to discuss the strategy that works best for them.
44	I can assist students with disabilities to manage and overcome trauma.
45	I can let students with disabilities to make choice and decide what works for them.
46	The school has an environment in which students feel empowered while dealing with trauma.
47	I know and support school policy on discrimination.
48	I can recommend or punish anyone who discriminate against students with disabilities.
49	I can contribute to policymaking on discrimination.
50	I know how to educate others to accept or embrace diversity.
51	I can promote the inclusion of traumatised students with disabilities in all school activities.
52	I can promote respect and empower students with disabilities in schools.
53	The school has a supportive environment with multicultural values to deal with trauma related issues.

Note: 1 = strongly disagree, 2 = Disagree, 3 = Neutral, 4 = Agree, 5 = Strongly agree.
